# Current Trends in Integration of Nondestructive Testing Methods for Engineered Materials Testing

**DOI:** 10.3390/s21186175

**Published:** 2021-09-15

**Authors:** Ramesh Kumpati, Wojciech Skarka, Sunith Kumar Ontipuli

**Affiliations:** 1 Department of Fundamentals of Machinery Design, Silesian University of Technology, 44-100 Gliwice, Poland; Ramesh.Kumpati@polsl.pl; 2 Department of Aeronautical Engineering, Marri Laxman Reddy Institute of Technology, Dundigal 500043, Telangana, India; opssk1995@gmail.com

**Keywords:** composites, engineering materials, acoustic, infrared thermography, nondestructive testing methods

## Abstract

Material failure may occur in a variety of situations dependent on stress conditions, temperature, and internal or external load conditions. Many of the latest engineered materials combine several material types i.e., metals, carbon, glass, resins, adhesives, heterogeneous and nanomaterials (organic/inorganic) to produce multilayered, multifaceted structures that may fail in ductile, brittle, or both cases. Mechanical testing is a standard and basic component of any design and fabricating process. Mechanical testing also plays a vital role in maintaining cost-effectiveness in innovative advancement and predominance. Destructive tests include tensile testing, chemical analysis, hardness testing, fatigue testing, creep testing, shear testing, impact testing, stress rapture testing, fastener testing, residual stress measurement, and XRD. These tests can damage the molecular arrangement and even the microstructure of engineered materials. Nondestructive testing methods evaluate component/material/object quality without damaging the sample integrity. This review outlines advanced nondestructive techniques and explains predominantly used nondestructive techniques with respect to their applications, limitations, and advantages. The literature was further analyzed regarding experimental developments, data acquisition systems, and technologically upgraded accessory components. Additionally, the various combinations of methods applied for several types of material defects are reported. The ultimate goal of this review paper is to explain advanced nondestructive testing (NDT) techniques/tests, which are comprised of notable research work reporting evolved affordable systems with fast, precise, and repeatable systems with high accuracy for both experimental and data acquisition techniques. Furthermore, these advanced NDT approaches were assessed for their potential implementation at the industrial level for faster, more accurate, and secure operations.

## 1. Introduction

Recent technology development in engineered materials has been focused in a wide variety of sectors anywhere from biomedical to aerospace applications. The components used in these sectors are diversified materials specially designed using various modifications to perform a desired function in engineering operations. These novel engineering materials are essential for applications that require internal, external, static, dynamic load, mechanical load, extreme temperature, and corrosive exposure. The material structure plays a pivotal role in the upkeep of mechanical characteristics. These specially designed advanced materials/structures exhibit greater strength, weight reduction, modulus, and other properties including safety. Furthermore, these engineered materials also demonstrate advancement in the manufacturing process, efficiency, quality, and cost-effectiveness. Production of these engineering components includes several specific procedures including use of multilayered or multifaceted synthesis where several varieties of imperfections/inadequacies may be introduced to finished products. These materials generally contain cracking resistance under lightweight stress conditions. However, periodic testing and detection is required for tectonic coherence, defect diagnosis, and safety analysis of the refined/finished/polished products in their functional environment. Two primary methods are used to assess engineered material microstructures i.e., indirect and direct techniques. Indirect techniques measure structural parameters i.e., grain size, using other material parameters. For example, the average particle size can be measured from periodic multiplication of the lattice parameter. Direct techniques measure the desired structural parameters directly by a measurement technique [1]. Engineered materials are classified into five essential groups—metals, polymers, ceramics, composites, and semiconductors. Semiconductors are specifically charged materials while the other four groups are either structural or charged materials depending on their applications. Irrespective of above said group of materials the important material properties are affected by geometrical array and bonding type of molecules/atoms. There are generally three types of bonds in engineered materials i.e., metallic, ionic, and covalent bonds. Metals and alloys are comprised of metallic bonds; semiconductors form covalent bonds, and ceramics have both ionic and covalent bonds [2].

The structure of designed materials relates to the organization of its individual components at the nuclear level where the structure is fixed according to the molecular organization. In crystalline materials, the atoms are coordinated in repeating clusters known as a lattice structure. Although there are numerous potential crystal structures, some of the more commonly observed structures in metals are face-centered cubic (fcc), body-centered cubic (bcc), hexagonal closed-packed (hcp), and tetragonal [2]. Numerous metals and their alloys exist in more than one crystal structure depending on composition and temperature, but the majority fall within these four gem structures. The engineering material structure can be categorized as macrostructure, microstructure, crystal structure, electronic structure, and nuclear structure. The microstructure of most specialized alloys for a given engineering application includes different stages that may contrast in their physical properties, setup, morphology, estimate, volume division, etc. Although the material choice for a particular application may focus on a particularly important useful property, often an auxiliary property such as solidifying extend, toughness, corrosion, creep resistance, or organic compatibility will play an overpowering part within the effective use of the material [3]. For engineered materials production using multiple material types (multi-components) in various layers (multi-layers), various angles (multi-facets) may be one of the failure causes for the material in the finished or operating state. These NDT methods are applying in the testing the quality of pipes, tubes, aerospace, automobile, storage tank manufacturing, in military and defense, and in nuclear industries. The above reasons necessitate discussing various advanced techniques enabling adequate diagnosis/testing of engineered materials to ensure they fulfil their desired technological applications with superior performance.

This scientific review facilitates the process to overcome the various problems encountered with engineered materials and can act as a bridge to enhance knowledge of nondestructive diagnosis tools. This paper reviews advanced nondestructive testing (NDT) methods for engineered materials. The main goal of this review is to identify material structural behavior such as shape, size, and mechanical properties that can affect the material purity/coherence, resulting in damage when external or internal forces are applied. Therefore, to avoid incalculable losses, the manufacturer or engineers must examine the material testing during the initial stages of the design process. During operational conditions, materials should be periodically inspected for their structural capabilities. Depending on the application, the material assessment process can include either destructive or nondestructive methods. Destructive methods result in change/damage to the shape, structure, arrangement of molecules, whereas NDT methods result in no damage/change to the material structure. NDT methods are typically preferable in manufacturing and functioning circumstances.

## 2. Review Methodology

This review provides a consolidated list of advanced NDT methods including their applications and unique advantages. The review methodology consisted of the selection and gathering of various research domains from various regional areas and several digital platforms. The selection benchmark contained four segments which will be sequentially discussed. In the first phase, significant keywords were defined to obtain details regarding specified domains and finally collect the results. In the second phase, the specified topics were verified, the excessive material omitted, and resubmission reviewed for the name and year of the published article and checked for relation with defined keywords. For the third phase, the abstracts were reviewed and to identify whether the research papers were relevant to NDT. The search selection was constrained to NDT techniques and non-relevant collections were removed. Selected papers were assessed, and the citations evaluated. In the fourth phase, collected papers were fully reviewed including the introduction, figures, tables, and discussion. At the end of the stage, the conclusion and contribution of work respective to nondestructive testing techniques was assessed. The review process contained three phases—review level 1, review level 2, and contribution.

For review level 1, selected papers were reviewed in more detail to set the review criteria regarding advanced NDT methods for engineered materials. In this stage, the focal topics were defined to critically explain NDT advanced methods. The primary NDT techniques were identified through the assessment of reference titles which led to the identification of relevant potential literature.

In review level 2, as per the review criteria, all the collected papers were reviewed and integrated. The review results were presented as state of art. The current NDT methods already in practice were assessed, allowing for identification of potential issues that can be remedied using more advanced methodologies, especially as they apply to development of hybrid materials. The conclusions drawn from this critical review are based on approximately 10 years of research work sampling. Finally, the prospects and development as a roadmap for nondestructive testing techniques for engineering materials were explained.

## 3. Survey on Various Nondestructive Methods

**Figure 1:**
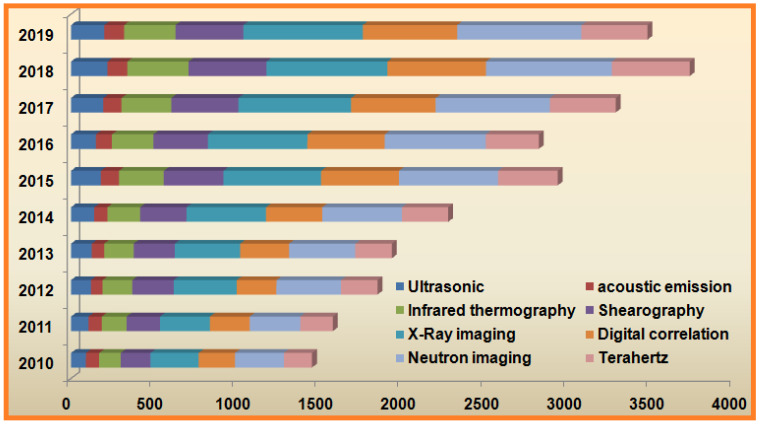
An assessment of publication numbers on various nondestructive methods and their applications for the last 10 years.

This review of advanced NDT methods for engineered material research over the past 10 years focuses on different aspects and general methods. During engineered material manufacturing, various defects or inadequacies may occur, making it imperative that the material quality and safety concerns during active performance be assessed [1]. The basis of nondestructive testing methods is to assess the quality of the structure without risk of damage to the specimen. Most legislative guidelines for NDT depend on the suggestion of autonomous worldwide organizations, such as ISO and ASTM standards. These autonomous organizations base their guidelines in portion on the investigation of producers and a few national and universal exchange affiliations. These exchange affiliations incorporate the Universal Committee for Nondestructive Testing (UCNDT), the American Society for Nondestructive Testing, and the Nondestructive Testing Management Association [4,5]. Comparing different types of NDT techniques can be troublesome; each method is interesting and outlined for its application. The chart below (Figure 1) supplies a wide range of various kinds of NDT tests over several years according to the principle type of technique. The year-wise number of publications on each particular NDT technique was consolidated, although the annual total number publications are also clearly enumerated in Figure 1. The advanced techniques i.e., terahertz, neutron imaging and digital correlation were the most mostly used NDT technologies.

Nondestructive testing explains the broad range of assessment and evaluation techniques for inspection of chemical and physical properties of material, component, and systems without any sample damage. These methods broadly include ultrasonic, X-ray radiography, neutron radiography, acoustic emission, nonlinear acoustic, infrared thermography, terahertz, shearography, and magnetic flux leakage testing. Nondestructive testing methods are routinely practiced in several industrial sectors including manufacturing of pipes, storage tanks, tubes [6], aerospace products [7], military and defense materials [8], nuclear industrials, automotives [9], stress testing in a rock bolt [10], biomedical materials, and composites [11]. As per the practice reported in a recent survey, NDT analytical tools are mainly terahertz, neutron imaging, digital correlation, X-ray imaging, and infrared imaging, and either one or more of these methods were typically used for inspection and evaluation of samples in a variety of industries. We found that in recent years several modifications were established on these sampling methods, type of analysis, and critical image analysis for the assessment of samples. Several developments were reported for NDT equipment including techniques, acquisition techniques, image processing, and computing power to upgrade practices.

In image-based techniques such as X-ray radiography, neutron radiography, micro-tomography, optical techniques, infrared thermography, terahertz testing, digital imaging, and shearography the upgrades and modifications were focused in the assessment of deformities, cracks and delamination, etc. The various NDT techniques along with their explained testing areas, applications, advantages, and disadvantages are tabulated in Table 1.

This review reports state of the art developments with respect to frequently used advanced NDT techniques used for assessment of engineered materials from a perspective paradigm. Engineered materials such as composites and components used in aviation, aerospace and automotive, and biomedical applications are evaluated using advanced NDT tests to examine the purity/quality, diagnose structural health, and estimate the residual life span under specific mechanical loading situations.

**Figure 2:**
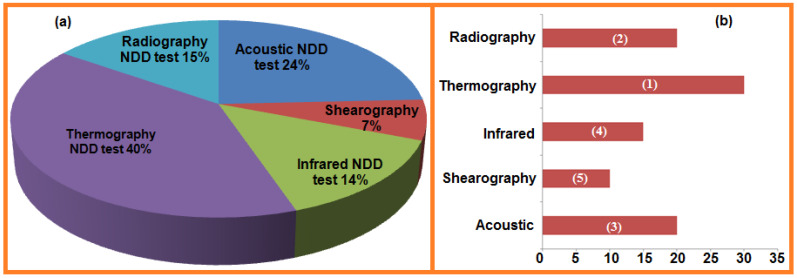
Recent development in the most practiced NDT techniques (**a**); number of papers reported and studied (**b**).

It should be noted that the nondestructive technologies used in assessment and evaluation of various engineered materials are reported for developments occurring over the past 10 years (2010–2019). Therefore, related published scientific research papers were found using several search engines with a significant increase in number regarding modifications and developments over the last decade. These reported research results and development of various modifications have caused an enormous increase in number of research papers on nondestructive testing methods, and this vast number of publications served as the basis for this critical review (Figure 2). Primarily, this paper describes the comprehensive parameters i.e., safety, cost, time and applications, along with their principle and uses. These parameters were taking into consideration for the comparative studies and described. Descriptions are also provided as necessary to discuss how material failure occurs in the production process, how one can assess material strength, and lastly how NDT methods can be used to evaluate material efficiency. Various reasons are noted for damage/defects of engineered material properties and their integrity. Secondly, NDT techniques are classified according to their application, and advanced NDT techniques are discussed from a case approach. Lastly, combined and simultaneous methods approached as per the application requirements will be explained.

### Failure Criteria of the Engineering Materials

Material failure hypothesis is the science of anticipating conditions under which strong materials will fail under the activity of outside loads. Essentially, engineered material failure criterion is distinguished as brittle (fracture) and ductile feature (yield). Material failure is dependent on different conditions such as temperature, stress, and internal or external load conditions. In many cases, engineered materials can fail in ductile, brittle, or both cases. Material failure criteria can be classified as macroscopic and microscopic failures. Bending moment failures, meanwhile, are often caused due to phenomenological and linear elastic fracture mechanical failure [12]. Composite polymers are widely used in railways, aerospace, and automotive industries owing to their resistance to harsh environment, bearable pay loads, and extreme mechanical properties. These composites may contain fibers, matrices, polymeric resin, and interfacial bonding in the micro- or macroscale [13]. These components may provide better mechanical properties, load bearing, and corrosive resistance. Composites commonly suffer from fatigue damage where sequential damage propagates throughout the matrix, producing cracking and debonding that can lead to major material failure. This delamination mechanism does not occur in polymer composites due to short fiber 3-D distribution [13]. Fiber breakage and delamination are considered high energetic damage while matrix cracks are least energetic damage [14].

There are several types of testing processes available in advanced composites or components for efficiency and safety evaluation. Material defects are the most common issues in composites and engineering components, and defect detection is crucial in the strategic maintenance of these structures. Defects that lead to malfunction of composite materials are composite cracking, fiber fracture, debonding, and pull-out [15]. Moreover, the NDT method is preferred for defect diagnosis without destruction and reduction of the diagnosis operational cost.

## 4. Classification of Nondestructive Testing

Nondestructive testing methods are typically used to examine sample strength and its changes over time [2]. NDT methods cover a wide range of analytical techniques for damage-free material testing. Nondestructive methods are divided into two groups—material contact and noncontact methods (Table 2). Both methods are applied at the time of material inspection. In many cases, the NDT methods rely on electrical sensors to collect accurate data. Noncontact material testing is used to collect specimen data while avoiding physical contact between the sensor and tested specimen or structure.

**Figure 3:**
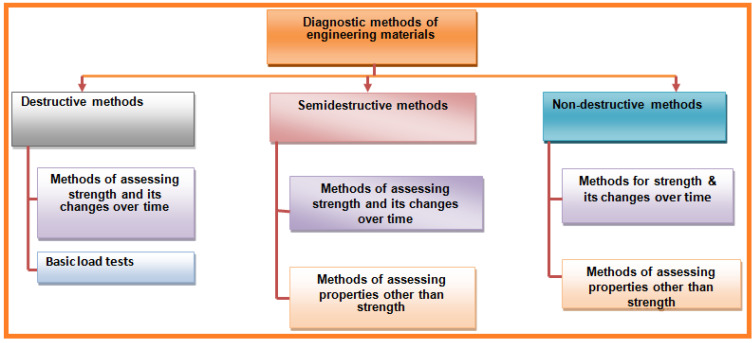
The basic classification of diagnostic methods for engineering materials.

Noncontact surface methods include ultrasonic, radiography, thermography, shearography, and physical inspection. Optical methods (i.e., thermography, holography or shearography) are among the more accurate noncontact methods [3]. The most common methods used for composite material testing are ultrasonic and radiographic methods. Appropriate method selection for material inspection is very complicated [3]. During development, cutting-edge demonstrative strategies are connected to materials and structural members. Another significant field is the nondestructive testing of engineering components/materials. Based on the degree of their intrusiveness, these methods can be categorized as destructive, semi-destructive, and nondestructive methods. General method classifications depending on principles and usage for diagnosing material are summarized in Figure 3.

**Figure 4:**
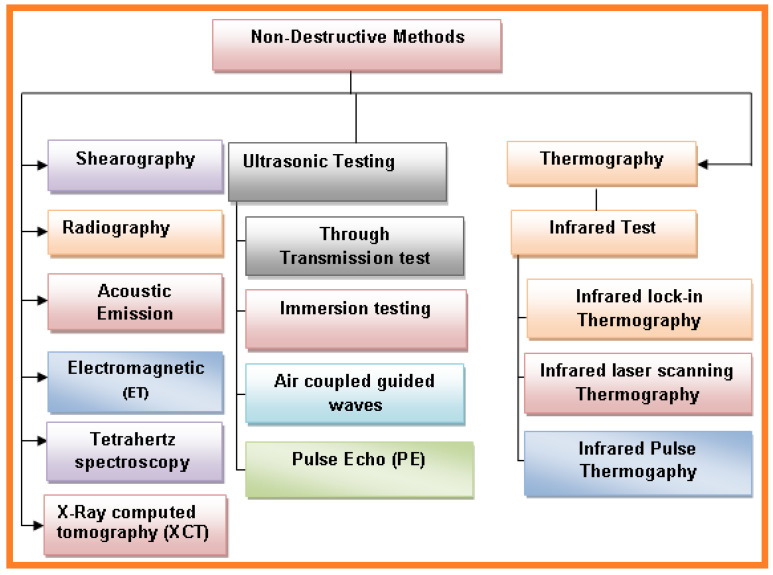
Most common advanced nondestructive diagnostic tests used for engineered materials.

For engineered material testing, a specimen part from the material structure or the entire structural member must be taken into consideration. Load testing must also be considered, along with semi-destructive and destructive test methods used to test the structural material for certain applications. Sometimes the material shape or size is distorted due to external or internal forces acting on the structural material. During operational conditions, the material crystallization can be change as well as other mechanical properties. These kinds of shifts in the material properties can ultimately affect the strength, size, or damage resistance. To avoid these issues, advanced mechanical tests are required for analysis. The most commonly used nondestructive tests are shown in Figure 4.

**Figure 5:**
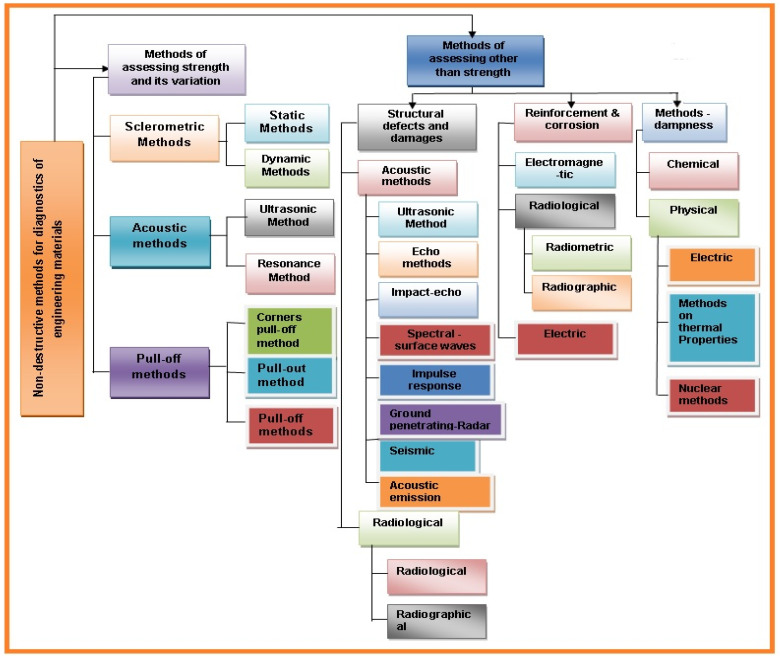
Nondestructive methods used for analysis of engineering material structures.

Additionally, in the case of nondestructive strategies, estimations can be repeated leading to confirmation and approval of the test [16]. All above methods are applicable for analysis of material characteristics and mechanical properties. The broad classification of nondestructive methods used for testing of material defects and mechanical properties are presented in Figure 5.

### 4.1. Shearography

**Figure 6:**
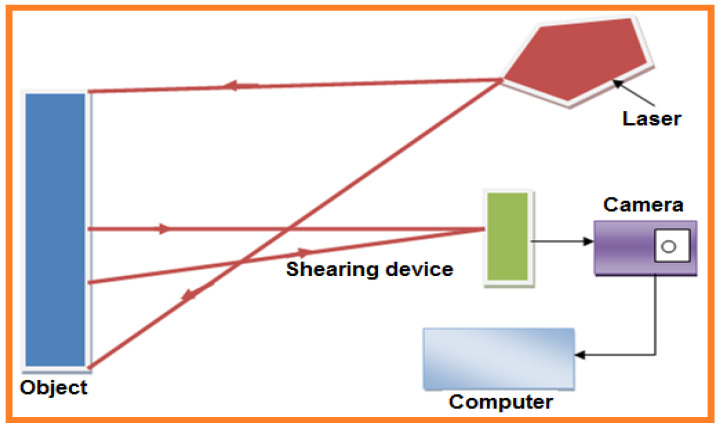
Line diagram of digital shearography for detection of cracks using laser point source.

Shearography testing is a noncontact laser-based technique [17]. The shearography method is used to detect material deformation (i.e., strain or displacement) [18] and has been improved to conquer holography limitations [19]. The applications are vast i.e., identification of defects and flaws [20], leakage, composite material delamination and scratching [21], displacement and material strain [22], curvature [23,24], residual surface stress [25], mechanical analysis [26], surface profile, and dynamic vibration. A shearography line diagram is depicted in Figure 6, where the test sample is imaged using an extended laser point source. An image-shearing device is fixed before the camera lens [19]. The image-shearing device brings two non-parallel rays of light scattered from two various sample points to intersect with each other.

The shearography method is best for composite material inspection and analysis of various composite parts (i.e., composite sandwich structures [27,28], composite pipes [29], wind turbine blades, aerospace and automotive structures including racing tires [30]). In aerospace technologies the most commonly used and preferred materials include polymer composites and sandwich core materials. The main disadvantage of shearography is in the characterization of matrix cracking and fiber debonding due to microscopic and mesoscopic damage mechanisms.

De Angelis et al. (2012) used [20] a laser-based numerical shearography method to detect and localize artificial defects in 4-mm-thick laminates (r = 15, 20, 35, 40 mm) at various depths (1.7, 2.5, 3, 3.2 mm). Delamination was detected using fuzzy neural analysis on approximately 30 samples within the same composite material. In another work, data accuracy was attained by double pulse illumination and stroboscopic laser [31]. Both spatial and temporal excitation methods are used for delamination examination because temporal phase modulation leads to noise reduction. The traditional laser shear speckle interferometry technique uses a withhold mode to obtain an interferometric fringe model that processes images acquired prior and afterward excitation. A few scientists have taken on phase shifting technology for improved image contrast. To accomplish this, the phase difference caused by object buckling is solved directly. The phase shifting technique improved image contrast by displaying the deformation distribution, which can then be quantitatively studied [32,33].An automated nondestructive inspection method that uses shearography-based thermal excitation to inspect a thermal insulation layer connected to a solid cylindrical structure has been developed [34]. By fabricating translation and rotation fixtures, this shearography approach can be used to inspect the whole surface of columns. Inspections are automatically scheduled by just entering the sample’s geometry, including length and diameter. To evaluate the inspection results, an image recognition technique based on deep machine learning has been developed, and an automated and orderly storage of the inspection results provides suitable management of the monitored data [34]. This technique is initiated to locate defects via the typical butterfly-type fringe model [34]. In 2019 Areco et al. developed [35] a new method for damage detection via cubic spline interpolation using differentiation of modal rotation fields. This method is achieved using speckle shearography, developed by Mininni et al. in 2016 [36] to lower uncertainty propagation and amplification. A cubic spline function was applied to the modal rotation field data at an adequate sampling interval to produce very smooth curvature profiles with a distinct damage signature. Furthermore, the amplitude of the defect signature in a given mode increases with the harshness of the damage.

In 2020, de Oliveira et al. used [37] lock-in check thermography and pulse shearography imaging for damage detection in composite fiber-reinforced polymer (CFRP) composite samples. For image acquisition, aEdevisOTvis 5000 system was used with FLIR SC5650 infrared camera containing 2.5 μm to 5.1 μm spectral sensitivity and 640 pixels × 512 pixels spatial resolution. The system used lock-in halogen lamp excitations with 1500 W halogen light sources. For shearography, the fixed aperture and wedge prism produced interference speckles using carrier fringes to separate interferometric phase information within the frequency domain. The system used a Pix-eLink camera (PL-D729MU) with CMOS sensor (3840 pixels × 2500 pixels), pixel size of 2.4 μm × 2.4 μm, and 0.05 s resolution. The applied image-processing tool depends on corner detection to correct buckling originated by lens imperfection. Image fusion also combined images from lock-in check thermography and square-pulse shearography. The fusion tools used include Boolean image fusion of binary images (BF), algebraic image fusion (AF), and principal component analysis fusion (PCAF). Another work performed by de Oliveira et al. combined [38] vibration, radiation, and induction procedures along with shearography on composites used in pipeline repairs for oil industry applications. After pipeline repair, damage extension was monitored using shearography combined with pressure, vibration, induction, and radiation. This work confirmed that the more attractive loading method uses shearography in NDT. In 2021, Anisimov and Groves developed [39] a high-speed shearography device for quantitative surface damage measurements on aluminum composite samples. To complete these measurements, the experimental data from in- and out-of-plane surface strain measurements of samples is coupled with numerical models. The setup contains two shearing interferometers symmetrically aligned in a horizontal plane with a gas gun as an impactor.

Generally, the shear amount adjustment and spatial carrier frequency are not completely independent. In some systems, both are adjusted by rotating mirrors, and the intensity of rotations necessary to attain a suitable shear amount and spatial carrier frequency fluctuate, reducing the precision of detection results. In 2020, Sun et al. developed [40] a spatial phase-shift-based defect detection shearography method using self-governing adjustment of shear amount and spatial carrier frequency. The shear quantity adjustment is achieved by changing the written image of a spatial light modulator (SLM). The spatial carrier frequency is controlled by the relative position between the dual apertures. Experimental results of both undamaged and damaged specimens indicate that this system is suitable for detection of internal and deformation defects.

In 2019, Katunin et al. developed [41] an NDT joint method using shearography and wavelet analysis. Both shearographic and wavelet analysis are thoroughly implemented to design the apparatus using simulated data with a shearography numerical model and further correlated with experimental data. This combined technique is very useful in plate-like compositions. Laser-continuous waves are illuminated on the plate surface with a 535 nm wavelength and passed via the acoustic optic modulator for improved vibrations on the plate. The produced intensity pattern by the interferometer is verified via digital camera with spatial resolution, temporal phase shifting, and shearing amount set to 10 mm. Three measurements of each of the modal rotation fields at different amplitudes of plate vibrations were performed. The amplitude of vibration is limited by looking at the raw fringes in a computer monitor.

### 4.2. Radiography Inspection Method

**Figure 7:**
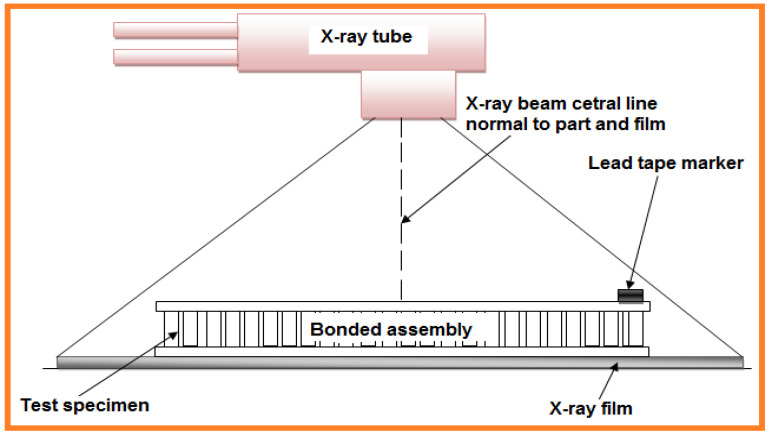
Radiography inspection method assembly for micro crack detection.

Radiography testing is an NDT method that uses either X-rays or gamma beams to inspect the internal structure of fabricated components by recognizing flaws or defects. In radiography testing the tested part is placed between the radiation source and film (or detector), a method used by many industries (Figure 7). Radiography methods are a popular NDT method since they competently identify various surface and inner discontinuities without many required accessory arrangements. In 2016, Hassen et al. [42] used X-ray radiography to examine embedded and composite texture manufacturing in glass fiber polypropylene composites. This radiography technique exhibited lower error than the phase array ultrasonic method, and it is predominantly used in aircraft engineering materials assessment. This method locates macroscale damages in laminated composites i.e., trans laminar cracks, including material delamination [43]. Crack detection analysis at the material surface [44] and location of damaged defects at the micro scale i.e., matrix cracking can be improved via dye penetration at the inspection site. This method is referred to as penetrate-enhanced radiography and applies to a variety of industries [45].

Radiographic inspection is regularly used for micro crack detection in strong composite laminates and broadly used to distinguish fiber breakage in honeycomb gatherings. Commonplace defects are observed in honeycomb gatherings are crushed core, center relocation, blown core, dimpled center, and intercellular debonding failures. In 2018, Yadollah et al. reviewed [46] material fatigue life in manufacturing materials, including a fatigue life forecast based on the crack distribution. This work concluded that the radiographic approach is an effective and reliable method for advanced material characterization.

### 4.3. Acoustic Emission Testing (AE)

**Figure 8:**
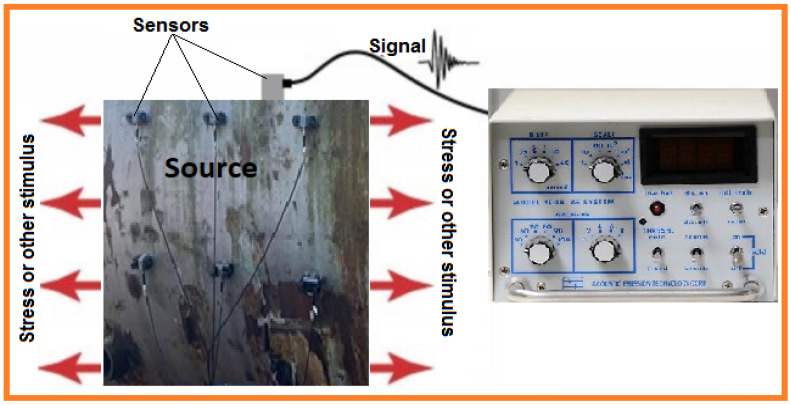
Schematic diagram of acoustic emission test (AE) for detection of material failure.

Typically, passive NDT methods depend on identification of brief bursts of ultrasound transmitted by dynamic splits beneath a stack. Sensors are scattered on the sample surface allowing the bursts and deformities to be identified by acoustic emission (AE) testing. It is indeed conceivable to identify plasticization in exceedingly focused regions using AE, even from recently split shapes. AE is routinely used in confirmation tests for pressure vessels, auxiliary wellbeing observing strategy on bridges, metal-matrix composites, spillways, and dynamic erosion detections. AE sample analysis is depicted in Figure 8. This test is mainly used to identify material fracture, fatigue, corrosion, and chemical changes within the material structural or oxidation. The major advantage of this technique is the ease and high accuracy for identification of imperfections, spalls, and cracks in engineered materials in industrial settings.

In 2013, Collini and Garziera proposed [47] the principle of acoustic as opposed to laser measurement, known as acoustic excitation–acoustic acquisition. Laser Doppler vibrometry was used to attain results via dynamic investigation using piezoelectric excitation. The experimental setup is comprised of a cylindrical shape loudspeaker with 125 mm wide cone and over 100 dB distortion. The setup also included a QSC RMX 2450 audio amplifier with 0.02% maximum distortion and Behringer ECM8000 microphones with multi-directional sensors (15–20 kHz frequency). Acquisition was executed by a multichannel device (Edirol FA101) with ten in/output channels (24 bit/192 kHz) with firewire interface. The Adobe Audition (version 1.5) integrated with ad hoc plugins analysis software was especially developed for NDT. In 2013, Connolly et al. applied [47] an acoustic wave technique with nonlinear modulation of ultrasonic pulses to detect cracks in complex grain structures. Acoustic wave traces are collected with applied fatigue and modulation loads for a set spatial propagation in each fatigue cycle. Ultra-scatterings were induced on the specimen to improve the signal-to-noise ratio via post-processing subtraction to achieve multi-dimensional image representation using ultrasonic time signals. The setup was comprised of a longitudinal wave transducer (5.0 MHz frequency) with wedges containing polystyrene linked to one end of the gauge length to produce surface waves. On the other side, a gauge length is mounted a wedge reflector connected to a monitored double-through-transmitted signal (84.1 mm distance from reflector to gauge length). The above setup and subtraction technique applied to these dynamic signals synchronized fatigue crack identification for real-time monitoring.

In 2013, Skłodowski et al. developed [48] an acoustic tracing method to assess detachment defects where the signals were registered by a video camera and FFT analysis of sound samples. Excitations and signals at dominant frequency were monitored with narrow band. In 2013, Cuadra et al. developed [49] a hybrid NDT method by incorporating heterogeneous monitoring containing a combination of acoustic emission, infrared thermography, and digital image correlation for the evaluation of fatigue loading and tensile strength on composite GFRP. The strain and temperature differential maps developed as hot spot defects in strain/load increments were correlated with divergent changes recorded in acoustic activity. Infrared thermography data quantified hysteretic fatigue measurements i.e., (i) heat energy dissipation, (ii) stiffness degradation, and (iii) average strain. To achieve this, a hybrid AE NDT set up containing a DiSP system (4 channel) and 2 piezoelectric transducers fixed on sample with cyanoacrylate adhesive transducers was assembled. The piezoelectric transducer frequency was 200–750 kHz along with 500 kHz pean, and the received signals were amplified using preamplifiers (2/4/6-AST) with a uniform gain of 40 dB. This hybrid NDT setup for glass reinforced polymer was evaluated using a combination of 3 methods i.e., acoustic emission, IRT, and DIC.

In 2014, Karabutov and Podymova quantitatively evaluated [50] CFRP composite materials via laser-ultrasonic spectroscopy (LUS). Microscopic delamination and voids on composite materials were detected by calculating the UTA (ultrasonic attenuation) coefficient. The observed resonance peak bandwidth depends on the porosity level due to imperfections (globular voids) and delaminated epoxy layers. Later in 2019, this group developed a combined LUT method for testing the porosity in particulates of reinforced metal-matrix composites fabricated by stir and insitu reactive casting techniques [51]. The effect of porosity dispersion by longitudinal acoustic waves was determined through broadband acoustic spectroscopy along with laser-ultrasonic spectroscopy. Aluminum-silicon alloy A336 matrix with reinforced SiC micro particles and insitu Al/Al3Ti reinforced with the Al3Ti intermetallic particles were studied. In both composites the phase-velocity dispersion and high velocity (i.e., 20–40 MHz) increases with reinforcement content independent of porosity. Similarly, at low frequency velocity (i.e., 3–10 MHz) porosity increase is independent of the reinforcement content. This study was the first to report the unified porosity-phase velocity functional relationship for particulate reinforced metal-matrix composites. The experimental setup used broadband acoustic spectroscopy with laser thermoelastic excitation and piezoelectric detection with ultrasonic pulses. A Q-switched Nd:YAG laser was used with a nearly Gaussian temporal profile with fixed pulse width (10–11 ns). A fixed optical system containing convex and concave lenses to form the laser beam achieved nearly Gaussian lateral intensity. This novel development proved the empirical relation between the porosity of the scanned composite and relative phase velocity dispersion can be described using a unified power function. This power function is considered the calibration curve for nondestructive quantitative evaluation of metal-matrix composites.

In 2019, Nakahata et al. developed [52] a photoacoustic microscopy (PAM) with combined confocal laser and ultrasonic techniques for rapid thermoelastic sample expansion. The interior/subsurface flaws were imaged in thin laminar (anisotrophic CFRP) material using an aperture-focusing method (SAF) applied with photoacoustic microscopy (PAM). The subsurface delamination was accurately (shape and location) estimated by the velocity distribution using an aperture-focusing method with improved photoacoustic wave amplitude. A compact Nd:YAG laser was used for laser light generation with 0.6 mJ pulse energy and 100 Hz pulse repetition (Nano L90–100, Litron, Palo Alto, CA, USA) able to emit 4 ns-long pulses with 532 nm wavelength. The external trigger signal was used for laser emission and reshaped using an axicon lens (83–781, Edmund Optics, Barrington, NJ, USA) to achieve a ring pattern. This ring pattern targeted on the sample via reflecting prism (OPL-PAM/532-CL-00, OPTO-LINE, Wilmington, MA, USA), then the released photoacoustic waves were detected using an UT transducer (V214-BB-RM, Olympus, Shinjuku, Tokyo, Japan). The data acquisition system was used to amplify and digitalize measured signals at 500 MS/s. This method offers vertical high-resolution images due to acoustic waves with wide frequency along with pulsed-laser irradiation.

In 2019, Li et al. used [53] multi-array transducers to initiate acoustic waves based on Snell’s law and APRT (acoustic pressure reciprocating transmittance). The inspection area was divided, and the wedge parameters (incident angles) were iteratively designed. A response simulation pattern of ultrasonic arrays was established based on FEM (finite element method), and an experimental process was established on the sample blade welded by linear friction. The incident deflection angle of beam ranges from 29–47°, producing strong acoustic transmission energy within the weld area. At the transducer position, the detected crack depth was simulated then analyzed. The results were clearly identified with high SNR, verifying the practicability and effectiveness. Finally, a scheme for damage inspection of the engine blade welded by LFW and other butt-welding surfaces of complex components with ultrasonic arrays was proposed.

In 2020, Abouhussien and Hassan assessed [54] damage sequences of crumb rubberized (0–30% content) concrete mixtures (SCRC) using acoustic emission (AE) through four-point flexural tests. AE sensors were fixed using epoxy adhesive for checking of damages by four-point flexural test. Piezoelectric sensors with integral preamplifier (R6I-AST) were used to achieve higher sensitivity, lower resonance (55 kHz) frequency. The AEDAQ (data acquisition) was processed via AEwin signal processing software with 40 dB amplitude. In 2020, Selim et al. proposed [55] a hybrid technique combining ultrasound with conventional transducers for damage examination of metallic components. The combined technique overcame all photonic and ultrasonic method obstacles while combining all the advantages of both methods. Ultrasound is produced by pulse laser to examine the sample from larger distances and enable sample scanning. The broad frequency excitation laser removes interferometer stability problems compared with ultrasonic transducers. This hybrid system enhances the dimensions of the size and location by carrying the scan dimensions on a specific area, known as the synthetic aperture-focusing technique (SAFT). SAFT can imagine the entire volume provided by a 2D or 3D image of the sample depending on successive sample scans and resolution. A Nd: YAG laser emitting 8 ns pulsed with at 532 nm wavelength and 10 mj/pulse energy was used. The laser ray initiates thermoelastic expansion, producing the stress wave, and the acoustic pulse produces broadband ultrasound, imaging the sample’s inner side. The laser beam was scanned in a specified sample area by programmable galvanometer. At each excitation point, the UT waves reflected off the sample are observed by the UT transducer. The ultrasonic sensors (Olympus V133-RM) linked with the sample surface at 2.2 MHz frequency. The sensors are mounted on the incident surface and front side, and the signals are sent to a preamplifier (Olympus 5662) and Gage A/D card (50 MHz sampling frequency, 16-bit resolution) connected to a computer for further data processing.

### 4.4. Electromagnetic Testing (ET)

**Figure 9:**
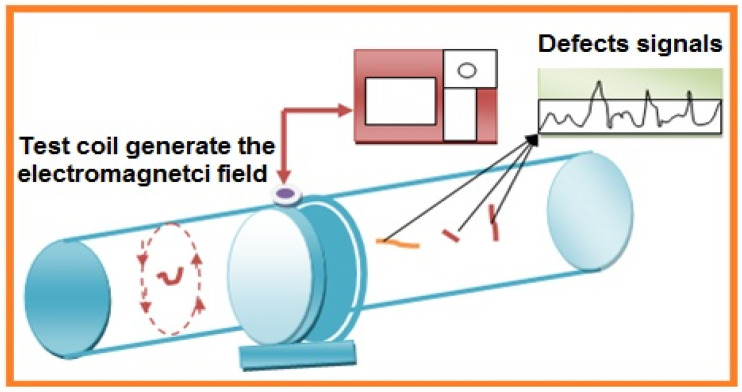
Schematic representation of experimental setup of Eddy current testing.

This testing method uses an electric current or magnetic field passed through a conductive sample. There are three sorts of electromagnetic testing (ET)—counting eddy current testing, alternating current field measurements (ACFM), and remote field testing (RFT). A schematic diagram is shown in Figure 9. Eddy current testing uses an alternating current coil to induce an electromagnetic field into the sample. Rotating current field estimation and farther field testing both present an attractive method, with RFT by and large used to test channels [56]. This test is generally used to detect sizing of metal discontinuities in terms of corrosion, wear, pitting, baffle cuts on the material, wall loss, erosion, and cracks in nonferrous engineered materials.

### 4.5. Ultrasonic Testing

Ultrasonic testing involves the transmission of high recurrence sound into a material and quantifies the reflected waves or transmitted waves. Rose (1999) [57] revealed the developments in tomographic ultrasonic imaging procedures that employed special features to replace the standard ultrasonic methods. The ultrasonic testing method is classified into pulse echo (PE), through transmission (TT), and time of flight diffraction (ToFD).

#### 4.5.1. Pulse-Echo (PE) Inspection Method

**Figure 10:**
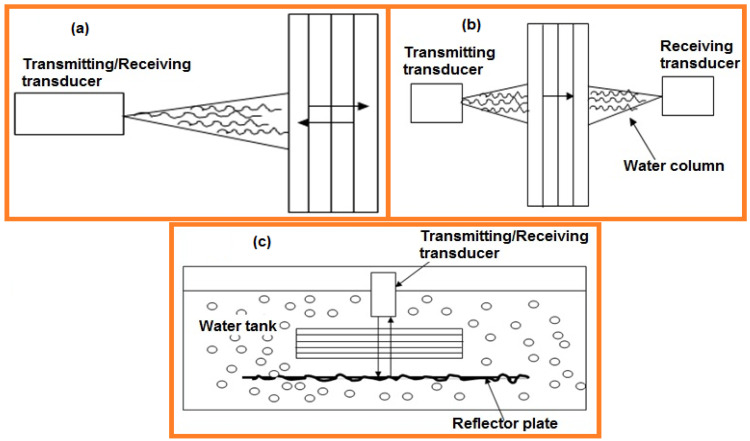
Schematic representation of experimental setup (**a**) pulse-echo inspection, (**b**) through transmission testing, and (**c**) immersion testing.

This method emits a sound beam into a test fabric surface. The sound travels through the raised divider portion of the fabric, and then returns to the transducer or return early when reflected from discontinuities inside the part. For a known acoustic velocity, the measured time interim at that point is used to derive separate paths within the material. These dispersed signals/waves have propagated along a waveguide composed by segments with different dispersive properties by processing the detected echo signal. Several researchers have dedicated to the research for propagation of signals for fast identification and separation of guided modes. De Marchiet al. (2013) [58] proposed the warped frequency transform (WFT) was applied to compensate the wave dispersion due to the traveled distance in a portion of the waveguide as a reference. Pulse-echo inspection method detects cracks, fissures, and other defects (Figure 10a).

#### 4.5.2. Through Transmission Testing

TT uses partitioned transducers to emanate and measure sound. The transmit test is situated on one side of the sample, and the get transducer is situated on the other side. As sound passes through the sample, it is constricted by internal features such as porosity. This procedure is often used to measure sample thickness (Figure 10b).

#### 4.5.3. Immersion Testing

The necessity to dampen ultrasounds during testing can be a challenge for huge or complex geometric tests. For immersion testing, samples are submerged in water, typically using a submersion tank. This strategy is typically improved using actuators that move the sample and/or setup inside the tank during ultrasonic inspection (Figure 10c).

#### 4.5.4. Air-Coupled Guided Waves

Ultrasonic-guided wave NDT strategy employs air (as opposed to water or gel) as a coupling medium to maintain a strategic distance of the sample from contaminations and harmful exposure, referred to as air-coupled guided wave NDT. Well-established routine air-coupled UT resists infiltration into strong materials due to high impedance at the air-solid interface that can be sorted using an acoustic mode change into guided waves. Further focusing a wave exactly on the target object is considerable problem when through the inhomogeneous medium. Fink et al. (1989) [59] solved the problem in acoustics first time by developing the time-reversal mirror (TRM) in the ultrasonic domain. For this they used piezoelectric transducer arrays. In this process, piezoelectric transducers properties were taken into considerations that are transmittance, receive capabilities, linearity and instantaneous measurement of the temporal pressure waveforms [60].

The elastic waves also can generate in air-coupled guided waves, but the huge disparity in acoustic impedance in between air and samples which leads to ineffective identification of impacts and potential damage evolution. Several researchers worked on the Structural Health Monitoring (SHM) approaches based onusing a set of piezoelectric transducers of guided elastic waves [61]. The set of transducers is used for the collection of the training data, but the smaller the variation in the localization performance was found. Recently, Miniaci et al. (2019) [62] developed a single piezoelectric transducer to achieve the enough training data for provided that the impact location with excellent accuracy. They proposed that the Lamb guided waves were excited by piezoelectric transducer on the sample for achieving impact-like time-history. The set of signals of the training data was collected are recorded at the out-of-plane velocity. Park et al. (2012) [63] used scanning laser Doppler vibrometer (SLDV) for automated and expedited training data that can measure out-of-plane velocity (due to Doppler Effect). The impact localization through acoustic and ultrasonic waves is generated by the impact event and surface-mounted transducers. They proposed that the impulse response functions excited through the surface-mounted lead zirconatetitanate (PZT) transducer and sensed by SLDV. Ciampa et al. (2017) [64] developed ultrasonic phononic crystal waveguide transducer that exhibits both single and multiple-frequency stopbands filtering out fictitious second harmonic frequencies. This sensing device can be easily fabricated and integrated on sample surface without alteration of its mechanical and geometrical properties; that attenuates second harmonics caused by the ultrasonic equipment.

These elastic waves can convert into a Lamb wave to small degree; instead of this these elastic waves can be generated by comb-type ultrasonic transducers which contain several tips spread at regular intervals. These comb-type transducers can also be fabricated in the form of matrix of ultrasonic transducers and can be placed with uniform distance which is equal to the wavelength. Piezoelectric/static elements or membranes were used for the development of air-coupled transducers for the controlled angle to make the perfect projection on the sample which is caused for selective excitation [65]. These installed sensors are permanently employed for decrease the variations upon the successive measurements and improves the sensitivity [66]. These air-coupled transducers facilitate the propagation direction along the structure of the samples, reduces the necessity of scanning and possible to inspect coated structures [67]. Rose, (2014) investigated the linear phased array transducer systems used in real-time biomedical applications i.e., medical imaging for differentiation of malignant to benign tissue [67].

### 4.6. Thermography Methods

**Figure 11:**
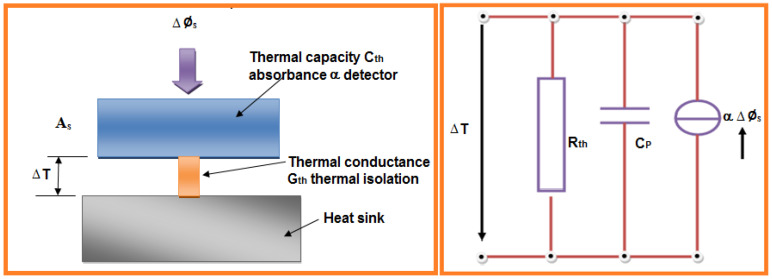
Thermo physical model of thermal infrared sensors in thermography technique.

The thermography method is an imaging procedure intended to use an object’s thermal radiation to calculate the material properties. Using Planck’s radiation and emissivity, one can calculate the surface radiation and temperature of the object. As per Planck’s radiation theory, every object emits radiation in the infrared (IR) spectral range at above absolute zero. By measuring this IR radiation, object radiation and temperature can be calculated without contact. Thermal and photon IR sensors can be used to detect the radiation. The schematic diagram with all components is shown in Figure 11. The emission of an object’s radiation depends on the radiance of the angle (Lambert radiator) and maximum quantity of energy emitted at a set wavelength. The spectral radiance, *L_λB_*, of an object is illustrated by Planck’s law (Equation (1)) [68]:

(1) $$L_{\lambda B}=\frac{2hc^{2}}{\lambda^{5}\Omega_{0}}\frac{1}{\frac{hc}{e^{kB}{}^{\lambda T}}-1}t$$

where *h* = Planck’s constant, *c* = the speed of light, *kB* = Boltzmann constant, *T* = Absolute temperature, Ω_0_ = unit solid angle (steradian). The subscript *B* = the relationship to a blackbody. For real radiators (bodies), it is necessary to multiply this equation by the wavelength-dependent emissivity, *ε_λ_*.

To calculate radiation fluxes, the spectral radiant exitance *M_λB_* is often required. This is the exitance given off into the half-space per surface area and per wavelength:

(2) $$M_{\lambda B}=\pi \Omega_{0}L_{\lambda B}$$

From Equation (1), Planck’s radiation is obtained:

(3) $$M_{\lambda B}=\frac{C1}{\lambda^{5}}\frac{1}{\frac{c2}{e^{\lambda T}}-1}$$

with radiation constants:

(4) $$C1=\;2\pi hc^{2}=3.741832\times 10^{-16}{\mathrm{Wm}}^{2}$$

The number of photons radiated per unit time, surface area and wavelength *Q_λB_*, and the relationship for the energy of a photon is:

(5) $$W=\;h\upsilon =\frac{\mathrm{hc}}{\lambda }$$

One can calculate the spectral photon exitance *Q_λB_* from Equation (3).

(6) $$Q_{\lambda B}=\frac{C1}{\lambda^{5}}\frac{1}{\frac{c2}{e^{\lambda T}}-1}$$

with the radiation constant

(7) $$C^{\prime }1=\;2\pi c=1.883634\times 10^{9}{\mathrm{ms}}^{-1}$$

With increasing temperature, the maximum radiation energy is displaced towards lower wavelengths, whereby the product of wavelength and temperature remains constant. This relationship is described in Wien’s Displacement Law [69]:

(8) $$\lambda_{\max}T=\;2898\;\mu m\;K$$

where the photon flux is $\lambda Q_{\max}T=\;3669\;\mu m\;K$

At the temperature of 23 °C, the maximum radiation wavelength is $\lambda_{\max}$ = 9.8 µm, or $\lambda Q_{\max}$ = 12.4 µm.

The radiant exitance MB in a particular wavelength range *λ*_1_ to *λ*_2_ is calculated by integrating over the corresponding wavelength range *λ*_1_ to *λ*_2_ from Planck’s Law of Radiation [54]:

(9) $$M_{B}=\int_{\lambda_{1}}^{\lambda_{2}}M_{\lambda B}d\lambda$$

The thermography method is an entrenched tool for nondestructive testing. This technique is using in military, industrial, medical, and civil engineering sectors. In 2001, Titman conducted [70] structural investigations related to building material, insulation, walls, and roofs using thermography testing. In 2015, Zhao et al. carried [71] out experiments on delamination depth, automated damage quantification, and degradation properties of composite materials. Additional tests are still required for different types of materials and damage to characterize their harm profiles via thermography, which is the key to creating the capability for recognition of various corrosion types in composites.

#### 4.6.1. Infrared ND Test

Infrared NDT alludes to the innovation using acousto-optoelectronics and electromagnetism to distinguish inner voids in materials or structures without harming their viability or quality. By measuring these spaces, the properties, condition, and quality of tested samples can be assessed [72]. As a modern NDT strategy, infrared thermography has the advantage of allowing for examination of large areas and delivering intuitive information rapidly and effectively [73].

Warm flow is related to quantifiable temperature scales, but it cannot be measured specifically in this manner. Heat flux is proportional to the thermal gradient *T*(*r, t*) in an object, the heat flow can be calculated by employing Fourier’s law describing heat flow interfaces [73]:

(10) q(r,t)=−k∇T(r,t)

where *q* (*r*, *t*) is the heat flux per warm flux per unit time on the isothermal surface within the course of temperature reduction, k is thermal conductivity (material), and ∆*T* (*r*, *t*) is temperature gradient.

Fourier’s law describes the relationship between warm flux and the temperature angle, and it is valuable for both still and moving areas. At that point, the differential condition of heat conduction can be used to show the internal relationship of temperature field with time:

(11) $$\nabla^{2}T(r,t)+\frac{q\upsilon }{k}=\frac{1}{\alpha }\frac{\partial T(r,t)}{\partial t}$$

where ά = *k/pc* is thermal diffusivity and *qv* is heat source.

From this, the hypothetical description of infrared warm imaging NDT can be analyzed by combining this equation with the boundary conditions. In terms of radiation, the concentrated radiation of a gray body is equal to the overall radiation intensity of a black body increased by the emission coefficient of the gray body. In other words, the radiation of a gray body is described by Stephen–Boltzmann’s law [74]:

(12) $$W=\varepsilon \sigma T^{4}$$

where *ε* is the emission coefficient of gray body, *W* is the Stephen–Boltzmann constant, and *T* is the radiation intensity, including the temperature of the material. Infrared thermography testing compares the relationship between thermal radiation and temperature.

**Figure 12:**
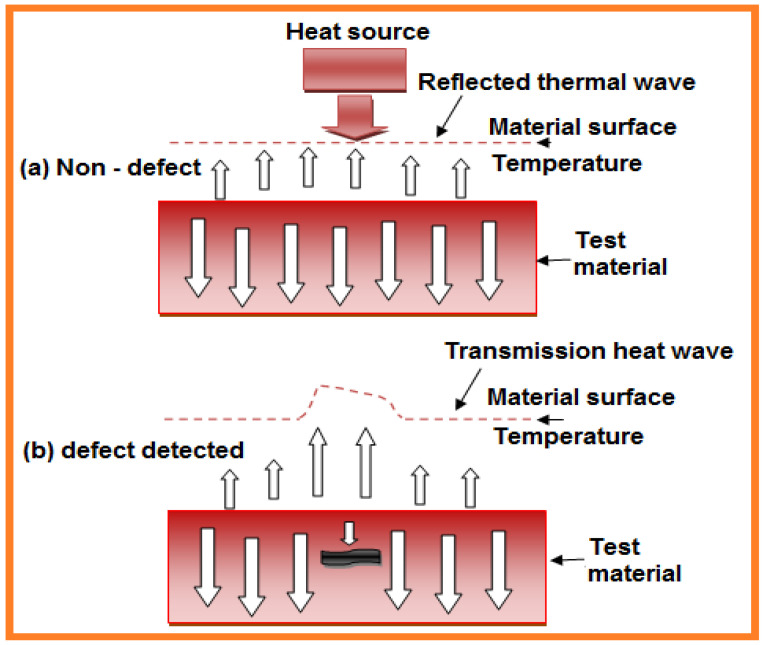
Schematic diagram of thermal wave imaging object detection: (**a**) non-defect; (**b**) defect detected object.

When the heat wave is contacted to the object surface, if the thermal wave propagates smoothly then the material is homogeneity. Eventually the thermal wave reaction on the sample surface reaches thermodynamic equilibrium. If defects are present within the specimen, reflection will occur where the heat wave moves towards the imperfection, producing sudden alterations in the surface temperature distribution (Figure 12). Infrared thermal wave NDT methods are divided into several groups.

In 2020, Qu et al. reviewed [75] special NDT techniques for detection of defects in advanced materials, elaborated test methods, and infrared thermal tests including various well-described NDT techniques. Some of the primary listed topics include infrared pulse thermography, infrared–lock-in check thermography, infrared ultrasonic thermography, infrared laser scanning thermography, and grating infrared thermal scanning. Referring to the existing research and application, infrared warm wave imaging is appropriate for distinguishing and screening of harmful material weaknesses such as cracking, rust, and debonding.

#### 4.6.2. Infrared–Lock-in Thermography Testing

Meola and Carlomagno (2014) used a lock-in module camera equipped with halogen lamp to create a sinusoidal thermal wave at selected frequencies for thermographic analysis on composite samples [76]. Direct wave surface interactions were measured using an oscillating interference model that measured the temperature and amplitude/phase angle, then presented phase images. This work confirmed that most NDT methods fails to detect the actual delamination based on reasons such as (i) tightly adhered multi-delaminated surfaces move the impactor away and (ii) delamination results in complex pathways. As a result, the composite sample delamination is often underestimated or undetected. Using a lock-in check module in IRT, sample damage is recognized by temperature rise from the extension of the directly monitored online warm area using time saving mode. In 2014, Meola and Carlomagno also detected glass/epoxy manufacturing defects using infrared thermography via the lock-in check thermography model [76]. This work showed the crucial role porosity and fiber alignment play in the generation and evolution of thermal signatures caused by initiation and proliferation of impact damage. In later works, Meola et al. (2015) suggested [77] that the infrared–lock-in thermography model along with the phased array ultrasonic methods was most suitable for precise damage assessment of the composite sample. This work also showed that lock-in check thermography and phased array ultrasonic methods are in satisfactory agreement, allowing for correct identification of defect size and location. This is achieved via integrated analysis of C-scan and S-scan phase images to provide useful information for excellent material evaluation. C-scans provide total view of the damage while S-scans exhibit damage location and phase images show directional-dependent fiber damage.

Several parameters are influenced during thermography including (a) thermal image system, (b) direction of heat flow injection, (c), heat source excitation, and (d) environmental factors. In the IR thermal imager system, influential parameters include (i) spatial resolution, (ii) temperature resolution, and (iii) frame frequency. The heat flow injection direction, as previously mentioned, directly affects the results. The heat source excitation, heating power, and time of excitation also influences the results, as the time is too short to cause sufficient conduction while long heating time adversely affects detection results. As a result, the appropriate heating power and time must be estimated according to the material type and actual conditions to ensure that successful defects detection. Environmental parameters that also directly influence the results include radiation, reflection, surface of the material and environmental convection of the signal [78].

In 2015, Vavilov et al. used IR thermography to inspect large aerospace composite parts without causing additional damage, avoiding excessive cost and time consumption [9]. In 2019, Deane et al. [79] combined pulsed thermography and vibro-thermography for automated real-time inspection of unmanned aircraft CFRP panels. Pulse heating was achieved by using 2 lamps (Balcar Xenon flash) at an energy of 6.400 J/flash with 2 ms duration at full width half maximum (FWHM). After sufficient time has passed, heat will travel throughout the entire sample and will decrease uniformly if the sample free from damage. On the other hand, if any defect/damage on the subsurface is present due to delamination/fiber breakage, an irregular temperature pattern is observed. This thermal response can be captured by infrared camera along with a synchronizer unit. Thesedata were stored as a 3D matrix at x and y spatial coordinates at time t. Active thermography was used by creating thermal contrast using external heating on the sample, causing inhomogeneities due to various thermal properties and diffusivity changes. These inhomogeneities are considered defects i.e., cracks, delamination, and debonding damages. Infrared cameras (FLIR Phoenix) with 640 × 512 pixels and 50 Hz data acquisition were used. These cameras collected IR photons that pass through the optics and are converted into electrons. After a set integration time, the charge was readout into a digital count, calibrated to temperature, then assigned to color or greyscale and ultimately presented as an infrared image. Data acquisition was completed using RDac from FLIR software while MATLAB and Ir_view from Visiooimage Inc. were used for signal processing. The primary development of this work is the advancement of processing methods using principal component thermography (PCT) and pulsed phase thermography (PPT).

Infrared pulsed thermography, infrared ultrasonic thermography, infrared laser scanning thermography, grating infrared thermal wave scanning and infrared image-processing technology are various modified techniques used for several applications. The optical flashlight is used in pulsed thermography for the surface inspection of the object and instantaneously heated such that heat penetrates the object [80]. If any defect is present on the subsurface of the object, the flow of heat is obstructed. Infrared ultrasonic thermography is also known as vibro-thermography where a vibration source is as a piezoelectric transducer, a booster, which amplifies the signal, and a sonotrode that transfer the signal to a test specimen. There is several stimulation approaches used in Infrared ultrasonic thermography may produces continuous, pulsed or modulated elastic waves [81]. In infrared laser scanning thermography, the high-power laser is a heat source in combination with pulsed thermography measurements (step scanning) or with continuous heating measurements. This work shows that laser line step scanning as well as continuous scanning both can be used within the developed super resolution techniques [82]. The integrated lock-in check techniquewas used to develop this method by Qu et al. (2019) without high frequency sampling to detect the horizontal and vertical cracks simultaneously [83].

### 4.7. Tetrahertz Spectroscopy

**Figure 13:**
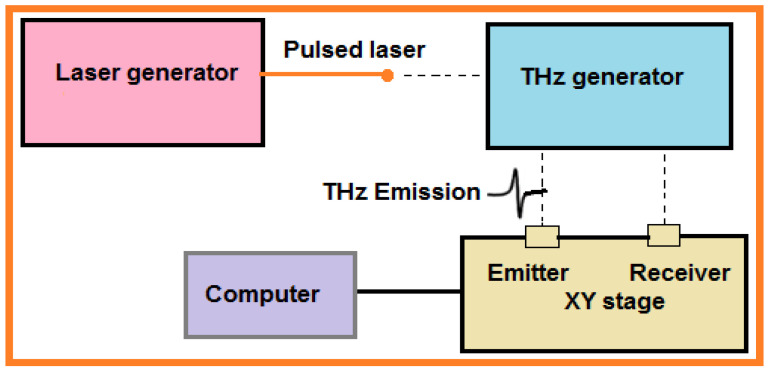
Schematic diagram of a THz generator and imaging system.

The tetrahertz (THz) region lies in the electromagnetic spectrum between 100 GHz and 30 THz, and this region overlaps with upper frequencies of the microwaves through the far infrared [84]. THz is low energy-based and can easily pass through nonmetallic and biological materials without causing damage. Recently, THz is used as a safe and precise non-damaging technique that can be used for imaging. Significant attention has been placed on THz for examination of the chemical makeup via molecular vibration and rotation interactions in condensed matter. Due to the unique properties of THz, it can be used as an NDT tool, a schematic diagram of which is shown in Figure 13.

In 2017, Kim et al. performed [84] a pulsed THz inspection on a GFRP material by adapting a XY imaging module. The THz waves were emitted as broad band width at a spectral array of 100 GHz to 3 THz for 20 fs resolution time. The device was connected with fiber optics, and the (SNR) signal-to-noise ratio was 60 dB, an acceptable power for fast and real-time examinations. The THz impulses were emitted and targeted on the sample using a set of mirrors. The maximum scanning in the XY imaging module was 150 mm × 150 mm with 50 µm resolution. In 2019, Ye et al. developed [85] THz time-domain spectroscopy (THz-TDS) to measure thermal barrier coating (TBC) thickness and characterized the TBC morphology after erosion. The 0° incidence angle was used in reflection mode for assessment of thickness before and after erosion. The thickness, refractive index, and internal structure evolution tendency of yttria-stabilized zirconia (YSZ) topcoat was estimated by contacting between pulsed THz waves and TBCs. Two mathematical models were developed to evaluate thickness loss and contrast results with the micrometer inspection method. In 2020, Cao et al. improved [86] and proposed the model-based method to measure the thickness of up to 4 coating layers on metal using reflected terahertz pulse echoes. This analytical model was formulated based on the Rouard method where the surface and interface coatings are rough as opposed to smooth. These properties were taken into account using the Kirchhoff approximation due to scattering when developing the model. Multilayer coatings were fabricated on the sample by stacking paper sheets on metal. The simulation showed that reflected THz signals decrease considerably due to scattering with increased surface roughness, but the interface roughness has only a slight effect on reflected THz signals.

### 4.8. X-ray Computed Tomography (XCT) NDT for 3D Printing

Three-dimensional (3D) Printing, also known as additive manufacturing (AM), is an advanced method to fabricate complex 3D components [87]. This process designs one layer on another using various nature of materials i.e., polymers, metallic pastes, composite, and ceramics according to the applications and requirements. Three-dimensional printing technology processes increased attention for creating solid models, patterns, prototypes, and stereolithography, and it is used for aerospace, automobile, electronics, and medical purposes. Furthermore, it minimizes manufacturing problems [88,89,90,91]. Generally, 1D and 2D technology was upgraded by several researchers, making advanced 3D concepts for complex components. The AM process generally contains several step combinations i.e., (a) conceptualization of CAD models, (b) conversion to STL format, (c) shifting to AM equipment and STL file manipulation, (d) machine setup, (e) object building, (f) removal and cleanup of the object, (g) object post-processing, and (h) application [92]. There are several types of AM procedures noted in the literature such as (a) photo-polymerization, (b) extrusion-based systems, (c) powder bed fusion, (d) material jetting, (e) binder jetting, (f) directed energy deposition, and (g) sheet lamination processes.

**Figure 14:**
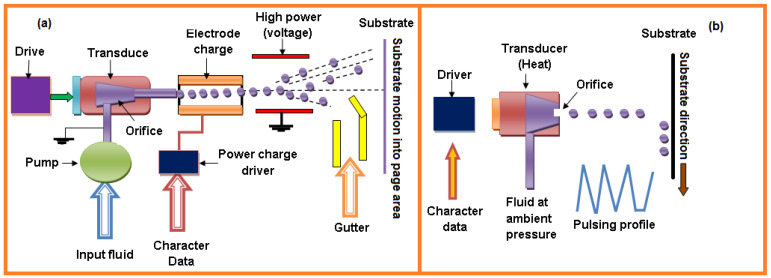
Schematic diagram of inkjet direct-write additive manufacturing process and continuous inkjet (**a**) and drop-on inkjet (**b**).

Developments and upgrades have led to hybrid processes known as hybrid and direct-write (DW) AM processes. These include (a) hybrid process, (b) direct-write AM techniques, (c) laser chemical vapor deposition, (d) plasma spray technique, and (e) inkjet printing technology. The new trend in 3D printing is the integration of two or more established manufacturing techniques into a single machine i.e., computer numerical milling and laser cladding have been combined with 3D printing to manufacture complex parts [93] which gives a novel, distinct setup that provides several synergistic advantages. Several novel hybrid 3D printing technologies have been reported in the literature, among which inkjet printing is most promising technology. Inkjet printing dispenses fluids at a rate of 1 MHz for continues droplets and 0–25 KHz per second for single droplets as required by the 3D material pattern. An inkjet printing process dispenses liquid metals, polymeric materials, and biomedical reagents. The fluid drops are produced as per the quantity of charge applied; if a continuously required charge is applied on the path (i.e., continuous inkjet technique) or where droplets are produced as needed by applying the voltage at a particular time (i.e., drop-on-demand inkjet technique). Various influential parameters in this technique include frequency, velocity, drop size, and printing sequence (Figure 14). Inkjet printing process applications include sensors, fabrication, electronics components design, semiconductors, medicine, prototypes, and pattern design [94].

**Figure 15:**
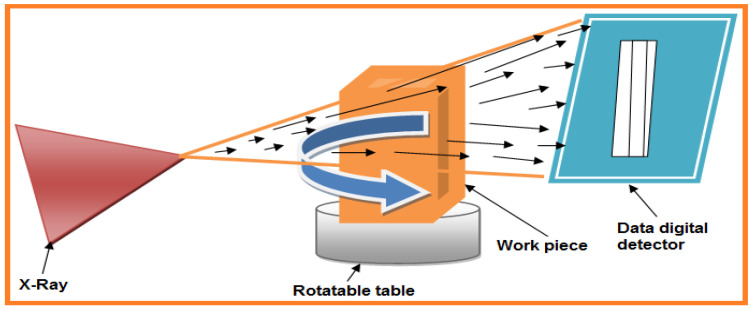
Line diagram of X-ray computed tomography (XCT) use in 3D printing technology.

In 3D printing technology, invalid printing and wrong process parameters can lead to improper porosity in prototype patterns, altered mechanical properties, and residual stress leading to ultimately deformation can occur. As a result, qualification and evaluation of 3D printed materials are necessary to avoid these deformities. NDT is required to characterize the material and defects, including some of the parameters listed such as cracks, voids, delamination, inclusion, structure, and porosity. Material-dimensional measurements are thickness, diameter, shape, mechanical properties, crack growth, and chemical compositions (metal alloy, phase definition, and impurities) [95]. The XCT method is a very accurate NDT for 3D materials. A schematic of XCT is represented in Figure 15 where the X-ray source, rotatable table (capable of 360° rotation in a clockwise direction), fixed work piece, and data digital detector are illustrated. The 2D photo projection of the work piece can be obtained from this method, where X-ray reduction of the workpiece is represented in terms of gray value. The entire experiment’s projection photos allow for complete rebuilding in terms of 3D division of the spatial X-ray reduction, ensuring a volumetric gray value main dataset of the entire area. The main advantages of this method include high accuracy, high X-ray energies, and sharp resolution. In 2020, Khosravani and Reinicke described a brief review of 3D printing and NDT evaluation techniques where they explain X-ray tomography as one of the most promising emerging NDT methods [96].

## 5. Application of Nondestructive Tests

NDT is broadly used for numerous vital world businesses. Any industry with expansive innovative material usage is likely to use a few kinds of NDT. Industries using NDT methods are listed in Table 3 below, along with inspection areas.

The above-mentioned industries use various material types such as polymers, metals, composites, plastics, thermoelastic, ceramics etc. NDTs are useful for detection of deformities, voids, cavities, debonding, delamination, and premature failures on these materials. X-ray computed tomography has been used in medicine [97], rock-like material fracture determination [98], bubble detection [99], geometrical verification [100], quantification of damage [101], and material science as a nondestructive technique.

Acoustic emission is regularly used for composite panels due to the compulsory space required to establish a network of sensors. Lamb waves are used for inspection of thin composite materials [102], macroscopic and delamination characterization, and elastic constant measurement [103]. Similarly, UT is widely used for composite materials irrespective of their anisotropy, which can be characterized using ultrasonic waves [104]. Infrared thermography is used for large-scale samples containing multilayered composite materials to detect temperature evolution. IT records variations in surface temperature against thermal and mechanical loads via infrared camera. IT also permits the evaluation of thermoelastic effects with linear temperature decrease in the elastic field. This can initiate generation of localized heat and global temperature of the material [105]. Recently used passive IT for evaluation of self-heating behavior reduces the time and cost of [106,107] experimentation.

In addition to the above applications, some researchers experimented with a combination of two or more techniques to achieve precise and accurate results with low cost. Some of the examples were explained regarding significant development in NDT techniques. However, in the application point of view, few works are explained as a combination of methods i.e., passive infrared thermography (IT), digital image correlation (DIC), and X-ray tomography were used to distinguish damage of CFRPs [108]. In 2016, Munoz et al. concurrently used AE, infrared thermography, and digital image correlation for damage detection in carbon/epoxy samples [109]. Similarly, in 2013, Cuadra et al. used combination methods (IT, AE and DIC) to quantify static and fatigue tensile damage of glass/epoxy composites [49]. Several researchers also reported the use of multiple nondestructive (AE, IT, UT, and passive and active Lamb waves) NDTs to attain accurate results on GFRP laminate samples [110,111]. This research proved that the combination of multiple nondestructive can be used to obtain an accurate diagnosis of the material damage. In this work we found that hybrid NDTs can be developed by combining multiple advanced techniques, which will ultimately ensure the safety and implementation of materials in engineering industries including biomedical as well as aerospace.

## 6. Significant Developments in NDT Tests/Techniques

Various developments and upgrades were observed in NDT techniques. The basic method for the IR technique can be described by heat diffusion into the inside of a material where the thermal and heat energy are modified by any abnormalities. Defects result in the appearance of hotter regions on the image. By processing this image, it is possible to efficiently characterize the damage. The infrared–lock-in thermography model along with the phased array ultrasonic method was most suitable to precisely assess composite sample damage. The reports also showed that the lock-in thermography and phased array ultrasonic methods reveal satisfactory agreement allowing for correct identification of defect size and location [77]. The upgraded lock-in module technique uses a camera equipped with halogen lamp to create sinusoidal thermal waves at specific frequencies for thermographic analysis of composite samples. This was achieved via direct wave interactions with the surface through an oscillating interference model that measured the temperature amplitude/phase angle and then represented phase images [73].

In advanced processing methods, the principal components of thermography (PCT) and pulsed phase thermography were used for defect contrast and visualization of the whole damaged area in UAV composite panels [112]. To use apixel-wise FFT tool in this image sequence, a heat flow adequacy photo and heat stream stage picture can be generated. The pictures of these two methods were fused for excellent damage detection in samples [37].

In the shearography, the main mechanism is the image-shearing device that causes two non-parallel rays of light scattered from two different object points to interfere with each other. The nearness of a deformity generates a periphery design within the interferometry phase difference map, which is the subtraction of an interferometric phase map after a stacking technique is performed. The developed setup contains two shearing interferometers (spatial resolution) symmetrically aligned in a horizontal plane with a gas gun as an impactor [39]. With the aperture arrangement and wedge prism, interference speckles using carrier fringes are produced, and the interferometric phase information in the frequency domain is separated [37]. Shear amount adjustment is also an excellent achievement attained by changing the written image of a spatial light modulator (SLM) [40]. The spatial carrier frequency is controlled by the relative position between the dual apertures.

The primary shearography sources include (a) radiation, (b) vibration, (c) induction, and (d) external force on the material [37]. In another work, cubic spline interpolation based on the differentiation of modal rotation fields was achieved using speckle shearography. Shearographic measurements and wavelet analysis are implemented to design the apparatus via simulated data with a shearography numerical model followed by further correlation with experimental data [41]. Several researchers have dedicated the time and knowledge to develop various NDTs that can be implemented in real life.

In AE NDT, the basic methodology involves scattered sensors over the structure surface to identify bursts and deformities. It is possible to identify plasticization using AE in exceedingly focused regions, including recently split shapes. The advantages of this testing arehigh accuracy, ease of use, imperfection identification, and early detection of imperfections.

The principle of excitation–acoustic acquisition involves the measurement of acoustics instead of laser interactions. Researchers used laser Doppler vibrometry where results were obtained through dynamic investigation using piezoelectric excitation [47]. Acoustic wave technique with nonlinear modulation of ultrasonic pulses was used todetect fatigue cracks in complex grain structures. Acoustic wave traces are collected with applied fatigue and modulation loads for set spatial propagation in each fatigue cycle. Another developed method is the acoustic tracing method used to assess detachment defects where acoustic signals were registered with a video camera and FFT analysis of sound samples. The excitations and signals at dominant frequency spectrum were recorded with narrow band components in a wider frequency [48].

A hybrid NDT method was developed by integrating heterogeneous monitoring techniques containing a combination of AE, infrared thermography (IRT), and digital image correlation (DIC) for the evaluation of tensile strength and fatigue loading on composite glass fiber-reinforced polymer [49]. Furthermore, a combined laser-ultrasonic method for porosity testing in particulate reinforced metal-matrix composites fabricated by stir and insitu reactive casting techniques [51] was also reported. A photoacoustic microscopy (PAM) with combined confocal laser and ultrasonic techniques for rapid thermoelastic expansion of samples was also reported. Interior/ subsurface flaw imaging in thin laminar (anisotropic CFRP) materials was detected using an aperture-focusing method (SAF) applied with photoacoustic microscopy (PAM) [52]. Several researchers developed these hybrid techniques or combinations of two or three NDT methods for more accurate and precise characterization of materials and safety establishment of engineered materials in practice.

## 7. Prospects and Development

In the many modifications and new developments discussed above, thermography systems are used for noncontact measurements in automated temperature control systems. Examples including, i.e., inspection of civil constructions and monitoring of chemical reactions, drying process, production of plastics and in automatic welding processes etc. Industrial image-processing techniques are used to analyze online data. Other developments can focus on the evolution of the novel model system. For example, in 2019 as reported by Ye et al. a vision-based system was developed that uses three different image-processing algorithms (gray pattern matching (GPM), color pattern matching (CPM), and average displacement tracking (MST)) for multi-point structural dynamic displacement measurement [85]. In the significant developments section, several examples were provided in detail. These developments show that thermography is still an emerging technology with many potential applications. In Table 4 applications, advantages and limitations of NDT methods are explained.

In this review, the authors found several examples of the combination or modification of methods and data acquisitions systems in the literature. These examples were reported in detail including references. These NDT developments provide more accurate measurements which lead to better and safer operations and practice. Future developments depend on intelligent, automated machine learning and deep learning to facilitate the rise of outstanding and potential techniques with improved accuracy and efficient information including image-processing algorithms. Several workers have proposed various automated diagnostic tests for fast, accurate, and precise analysis. However, some of these techniques are restricted to only certain material types, making them unsuitable for some applications. Despite existing NDT achievements, there is an essential need for notable research work to develop affordable systems with fast, accurate, precise, and repeatable systems for both experimental and data processing techniques. Further advancement of these methods is needed to implement NDT methods at the industrial level for better, accurate, and safe operations and practice.

## 8. Conclusions

Generally, many industries use different types of NDT tests depending on their applications. However, to detect material behavior, structural fatigue, size, and shape of the components before going to failure, material inspection is required under certain conditions. Currently existing NDT techniques are not suitable for all engineering materials. To address this issue in early stages, engineers must consider material testing. To accomplish this, several hybrid intelligent techniques along with excellent image-processing technology is needed. The described inspection strategies can be used in industry to avoid undetected damage and maintain a safe environment. In aerospace applications, low energy dynamic loads are essential, along with safety and operational reliability because these materials are bearing loads while undergoing fatigue without noticeable surface changes. Nevertheless, quantitative and qualitative identification of potential internal structural discontinuities after low energy impact is possible by means of ultrasonic and thermography NDTs. Infrared thermography, shearography, X-ray tomography, and ultrasound are excellent and mostly used techniques in industries. Tetrahertz and X-ray tomography can detect internal defects easily and can achieve excellent work potential in the future.

Hybrid technologies with one or more existing advanced method are combined with digital imaging and 3D printing technology may be the answer to the aforementioned issues in the future. Advanced NDT techniques are summarized in this paper along with their advantages and applications. This paper suggests that the further research is needed to combine/develop two or more nondestructive tests for better, cheap and rapid performance with accurate results. Furthermore, there is a need to develop existing technologies supplemented with intelligent automated machine learning and deep learning along with sophisticated algorithms for image processing.

## Figures and Tables

**Table 1:** Review survey on NDT testing methods with their applications.

NDT Methods	Testing Area	Applications	Advantage	Disadvantages
Acoustic emission (AE)	Cracking, debonding and delamination	Composite, fiber materials	Easy detection of fatigue racks, fractures, interface debonding, microcracks in matrix and delamination	Time consumed in data processing, required skills and experience in distribution of amplitudes in overlapped areas.
Ultrasonic (UT)	Material surface and internal defects	Elements, nonmetals, forging material and glued joints	Easy to detect, precession to find defects and adaptable defect area	Testing process on complex objects is complicated and more process time.
X-ray	Internal material defects	Material casting, non-metal parts and composites	Material defects i.e., porosity, slags, material abnormal penetration	Crack finding is not possible in perpendicular axis, not possible to find depth of crack, on-site online detection and the cost also high.
Eddy current	Material surface and small defects	Electrically conductive material	The operational equipment is advanced technology, testing object surface is not required to clean and less time to complete the test	Delicate in signals owing edge effect, suddenly alter, easy to allow the wrong display.
IRT	Calculated damage thickness, interlayers, and surface	Metallic and Nonmetallic materials	Noncontact of testing object, working area is large	To detect the material defect depth, complex algorithm is required such as mathematical calculations.
Magnetic Particle	Material surface and small defects	Ferromagnetic materials	It is low cost, portable and subsurface defects also detected	Restricted to ferromagnetic materials.

**Table 2:** A brief classification of contact and noncontact techniques for diagnosis of materials.

Material Contact Methods	Noncontact Methods
Magnetic testing	Through transmission ultrasonic
Eddy current testing	Radiography testing
Penetrate testing	Thermography
Electromagnetic	Shearography
Penetrate testing	Holography
Liquid penetration	Infrared Testing
Traditional ultrasonic methods	Visual inspection

**Table 3:** Types of industrial sectors using nondestructive testing methods.

Types of Sectors	Types of Test	Issues and Inspection Areas
Aviation	Ultrasonic test, Eddy current	Aerospace loads, cracking
Automotive	Ultrasonic test, Eddy current	Cracks, flaws inappropriate warm treatment and unsatisfactory fabric blends
Rail	NDT tests	Tracks, Automobile parts
Gas and other useful oil	NDT tests	Welds, tubes risers, tanks, and expansive forgings
Power Generation	NDT tests	Detect flaws in the turbines, tubing and related system
Manufacturing	NDT tests	Detect composite material defects and mechanical properties
Marine	NDT tests	Turbine blades and all other related powerplant parts
Military	NDT tests	Automobile vehicles related to military equipment
Utilities	NDT tests	Wastewater plants, tanks, pipes and home application

**Table 4:** Applications, advantages and limitations of the advanced Nondestructive testing methods used for damage detection.

Types of Test	Advantages	Limitations	References
Ultrasonic test	Suitable for various materials, able to locate internal flaws, compact, portable equipment can be used for on-site inspection	Complex setup, need skills to operate, Resolution is limited by algorithms and Computing power	[113,114]
Infrared Thermography	Non-ionizing radiation, realtime, suitable for wide materials, allows one-sided inspection, safe and easy to operate and cost-effective	Vulnerable and sensitive for insitu and field tests, limited by cost and availability of excitation sources, low accuracy with complex geometries, time consume for data processing depends on computing power and algorithms	[115,116,117]
Acoustic Emission	Provide real-time monitoring on growing flaws, highly sensitive to stress waves, suitable for insitu and field tests and it can cover large measurement volumes	Specimen must be stressed, sensitivity is affected by surrounding noise, not suitable for thick samples, difficult to interpret and characterize damage modes	[118,119]
Shearography tests	Noncontact and full-field surfaces train measurement More resilient to environmental disturbance Suitable for large composite structures Efficient for high-speed, automated inspection in production environments	External excitation sources are required Limited tolerance to rigid-body motion Limited capabilities for subsurface damage detection Accuracy depends on various sources of uncertainties	[19,120]
Tetrahertz tests	Robust and repeatable, high scan rate with imaging High precision, sensitivity and resolution High penetration depths for most composites Non-ionizing radiation Noncontact and full-field surfaces train measurement	Low speed of examination Restricted to nonconductive materials Costly	[121,122]
X-ray-tomography tests	Suitable for various materials and in situ tests Can detect both surface and bulk defects 2D and 3D images reveal the very Detailed shape of defects Special resolution at sub-micron level High efficiency	Not suitable for large size structures Not suitable for in-field tests Access to both sides required Dangerous ionizing radiation, requires protection Facilities and access are limited	[123,124]

## Data Availability

Not applicable.
